# Complete Genome Sequence of *Raoultella ornithinolytica* Strain B6, a 2,3-Butanediol-Producing Bacterium Isolated from Oil-Contaminated Soil

**DOI:** 10.1128/genomeA.00395-13

**Published:** 2013-06-27

**Authors:** Sang Heum Shin, Youngsoon Um, Jeong Hun Beak, Sehwan Kim, Soojin Lee, Min-Kyu Oh, Young-Rok Kim, Jinwon Lee, Kap-Seok Yang

**Affiliations:** Macrogen Inc., Gasan-dong, Seoul, Republic of Koreaa; Clean Energy Research Center, Korea Institute of Science and Technology, Hawolgok-dong, Seongbuk-gu, Seoul, Republic of Koreab; Department of Chemical and Biomolecular Engineering, Sogang University, Seoul, Republic of Koreac; Department of Chemical and Biological Engineering, Korea University, Seoul, Republic of Koread; Institute of Life Sciences and Resources & Department of Food Science and Biotechnology, Kyung Hee University, Yongin, Republic of Koreae

## Abstract

Here we report the full genome sequence of *Raoultella ornithinolytica* strain B6, a Gram-negative aerobic bacillus belonging to the family *Enterobacteriaceae*. This 2,3-butanediol-producing bacterium was isolated from oil-contaminated soil on Backwoon Mountain in South Korea. Strain B6 contains 5,398,151 bp with 4,909 protein-coding genes, 104 structural RNAs, and 55.88% G+C content.

## GENOME ANNOUNCEMENT

2,3-Butanediol is a very useful chemical, which is used as a solvent and as a precursor to various chemicals and synthetic polymers (1, 2). A 2,3-butanediol-producing bacterium, strain B6, was isolated from an oil-contaminated soil sample collected from the Backwoon Mountain in South Korea for screening of organic compound-tolerant bacteria. This strain was identified as *Raoultella ornithinolytica* by 16S rRNA gene sequencing and physiological analysis (3). The results showed the highest 16S rRNA gene sequence identity, 99.9%, to strain JCM6096^T^ (NCBI accession number AJ251467) (4). *R. ornithinolytica* (formerly known as *Klebsiella ornithinolytica*) is a Gram-negative aerobic bacillus which belongs to the family *Enterobacteriaceae*. *R. ornithinolytica* B6 can be grown using glycerol, hexoses (glucose, fructose, galactose), pentose (xylose), or disaccharide (sucrose) as a sole carbon source. When sugars are supplied, the main product of the bacterium is 2,3-butanediol, even at a relatively low temperature (25°C). Hence, *R. ornithinolytica* B6 has energy-saving advantages for industrial production of 2,3-butanediol.

The genomic DNA was isolated from an overnight culture of strain B6 using a DNeasy blood and tissue kit (Qiagen) and was sequenced by 454 GS FLX titanium pyrosequencing (Roche), following the manufacturer’s instructions, with 25× coverage (5). Subsequently, the 398,549 reads generated, with a total length of 126,825,580 bp, were assembled using a GS *de novo* assembler (version 2.3; Roche). The contig *N*_50_ was 145,738 bp, and the largest contig assembled was 438,809 bp. This assembly generated 86 large contigs (>500 bp), with a length of 5,365,416 bp. The 385,542 reads (96.73% of the total) were assembled into 9 scaffolds, which were composed of 67 contigs, with a length of 5,379,360 bp. The scaffold *N*_50_ was 3,097,984 bp, and the average length of the scaffolds was 3,097,984 bp.

The order of scaffolds was determined by a similarity search among published references and confirmed by a PCR size check. The gaps both within and between scaffolds were closed by long-range PCR (using MG *Taq*-HF DNA polymerase; Macrogen, Seoul, South Korea) and subsequent Sanger sequencing using an ABI 3730XL capillary sequencer. The sequences from ABI 3730XL sequencing and scaffolds were completely assembled into one circular genome using Phred/Phrap/Consed software (6) and annotated with the Prokaryote Genomes Automatic Annotation Pipeline (PGAAP) (7).

The complete genome of B6 is composed of a circular chromosome of 5,398,151 bp (55.9% GC content), which includes 4,909 coding genes, 79 tRNAs, and 25 rRNAs. Overall, 4,070 coding genes (82.90% of the total coding genes) have putative functions assigned on the basis of annotation.

A transcriptome analysis of B6 using the Roche/FLX system was performed to improve its genome annotation. Although small amounts of non-rRNA sequences were obtained from transcriptome analysis, 6,195 reads (91%) of the 6,746 mapped non-rRNA reads were mapped to 1,169 predicted coding genes.

We found that acetoin production increases rapidly in stationary phase in producing 2,3-butanediol. Also through transcriptome analysis, we found that the expression level of acetoin reductase increased sharply after 2,3-butanediol production. In producing 2,3-butanediol, it is necessary to block the route of succinic acid, lactic acid, and fumaric acid, as well as inactivate acetoin reductase.

### Nucleotide sequence accession number.

The complete genome sequence of *Raoultella ornithinolytica* B6 has been deposited in GenBank under accession number CP004142.
